# Therapeutic Electromagnetic Field (TEMF) and gamma irradiation on human breast cancer xenograft growth, angiogenesis and metastasis

**DOI:** 10.1186/1475-2867-5-23

**Published:** 2005-07-26

**Authors:** Ivan L Cameron, Lu-Zhe Sun, Nicholas Short, W Elaine Hardman, C Douglas Williams

**Affiliations:** 1 Department of Cellular and Structural Biology, University of Texas Health Science Center at San Antonio, 7703 Floyd Curl Drive, San Antonio, Texas 78229, USA; 2 Pennington Biomedical Research Center, Louisiana State University, 6400 Perkins Road, Baton Rouge, Louisiana 70808, USA; 3 EMF Therapeutics, Inc., P.O. Box 679, Signal Mountain, Tennessee 37377, USA

**Keywords:** electromagnetic field, breast cancer, ionizing irradiation, angiogenesis, metastasis

## Abstract

**Background:**

The effects of a rectified semi-sinewave signal (15 mT amplitude, 120 pulses per second, EMF Therapeutics, Inc.) (TEMF) alone and in combination with gamma irradiation (IR) therapy in nude mice bearing a human MDA MB231 breast cancer xenograft were tested. Green fluorescence protein transfected cancer cells were injected into the mammary fat pad of young female mice. Six weeks later, mice were randomly divided into four treatment groups: untreated controls; 10 minute daily TEMF; 200 cGy of IR every other day (total 800 cGy); IR plus daily TEMF. Some mice in each group were euthanized 24 hours after the end of IR. TEMF treatment continued for 3 additional weeks. Tumor sections were stained for: endothelial cells with CD31 and PAS or hypoxia inducible factor 1α (HIF).

**Results:**

Most tumors <35 mm^3 ^were white but tumors >35 mm^3 ^were pink and had a vascularized capsule. The cortex within 100 microns of the capsule had little vascularization. Blood vessels, capillaries, and endothelial pseudopods were found at >100 microns from the capsule (subcortex). Tumors >35 mm^3 ^treated with IR 24 hours previously or with TEMF had decreased blood vessels in the subcortex and more endothelial pseudopods projecting into hypoxic, HIF positive areas than tumors from the control group. Mice that received either IR or TEMF had significantly fewer lung metastatic sites and slower tumor growth than did untreated mice. No harmful side effects were attributed to TEMF.

**Conclusion:**

TEMF therapy provided a safe means for retarding tumor vascularization, growth and metastasis.

## Background

In a previously published experimental research report, it was found that exposing a transplantable murine mammary adenocarcinoma to a 15 mT EMF given at 120 pulses per second for 10 minutes per day significantly reduced tumor growth and vascularization and resulted in an increased survival time [1]. This published report appears to be the only literature available on the use of pulsating magnetic fields to reduce tumor angiogenesis. The authors of this report suggested that the magnetic field treatment used acted to reduce tumor angiogenesis and might have value as an alternative therapeutic modality for treatment of patients with tumors. The study reported here was designed to further investigate the potential of the same EMF therapy to inhibit growth and angiogenesis of a human breast cancer xenograft and to compare the effects of: 1) a commonly used course of gamma irradiation (IR) involving exposure to 200 cGy every second day for a total of 800 cGy, 2) daily exposure to TEMF, and 3) a combination of these two therapeutic treatment regimens on tumor growth, tumor angiogenesis, tumor metastasis, and of the side effects of each treatment regimen. Although this study used whole body IR therapy, most IR therapy of human patients is restricted to localized targeted regions of the body to avoid general side effects of IR treatment. The MDA MB231 cancer cell line transfected with and expressing a green fluorescent protein (GFP) gene was used to facilitate study of metastases of cancer cells from the site of the primary tumor [2].

Our study results demonstrate the potential of TEMF therapy to retard tumor: growth, angiogenesis, and metastasis, without harmful side effects.

## Results

### Body Weight

**Figure 1:**
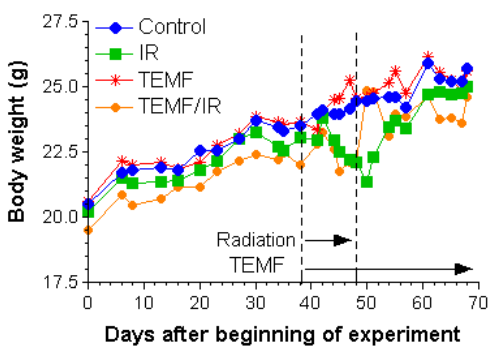
Body weights during the course of the experiment. The two groups of mice that received gamma irradiation both lost body weight during and for a few days after the course of exposure, but the body weights later recovered towards the weights of the two groups of mice not exposed to gamma irradiation.

Once the mice were divided into treatment groups the body weight of each mouse was measured every 3 to 4 days for the remainder of the experiment. As illustrated in Fig. 1, the two groups that received IR therapy every second day for 8 days demonstrated a mean body weight loss beginning during IR therapy and lasting until about 8 or 9 days after the end of IR therapy. After completion of the IR therapy, the irradiated mice again began to regain their weight toward the mean weight of the two groups of mice not subjected to IR therapy. The group of mice that received only EMF therapy demonstrated a continuous increase in mean body weight similar to the group of mice given no therapy.

### Tumor Growth

**Figure 2:**
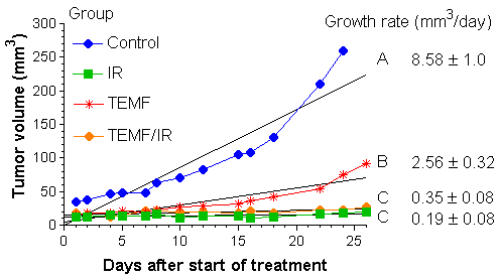
Illustrates the effect on tumor growth of: the 8-day course of gamma irradiation therapy (IR), the daily exposure to the TEMF therapy (TEMF), a combination of gamma irradiation and TEMF therapy (TEMF/IR), and no treatment (Control). The tumors were all 35 mm^3 ^or less at the start of treatment. The growth rate is the slope of the linear regression of the tumor volumes at each time. It can be seen that tumor growth was beginning to deviate from the linear fit about 18 days after the start of TEMF or radiation treatments however there was still a reasonable linear fit. ANOVA was used to determine any statistical differences in growth rates between treatment groups. Different letters demonstrate significant differences. Gamma irradiation alone (IR) or in combination with TEMF (TEMF/IR) reduced the tumor growth. The TEMF therapy resulted in a slower tumor growth rate than the untreated controls but not as slow as the gamma irradiated mice.

Fig. 2 illustrates mean tumor volume change for each of the four treatment groups starting at the beginning of IR and/or EMF therapy. All tumors in each therapy group were less than 35 mm^3 ^at the start of treatment period. To statistically assess tumor growth rate, the data on each tumor in each group of mice was subjected to linear regression analysis. Tumor volume gave a good fit to a linear regression model. The slope (growth rate) derived from the linear regression of each tumor volume was used to determine any statistical differences in growth rates between treatment groups (Fig. 2). Growth rate of tumors from the untreated group was significantly faster (p < 0.001) than any of the three groups of treated mice. The tumor growth rate of the group of mice treated solely with EMF therapy was significantly less than the tumor growth rate of the untreated group. The tumor growth rates of the two groups of mice treated with gamma irradiation were significantly less than the tumor growth rates of the untreated or TEMF only treated groups.

**Figure 3:**
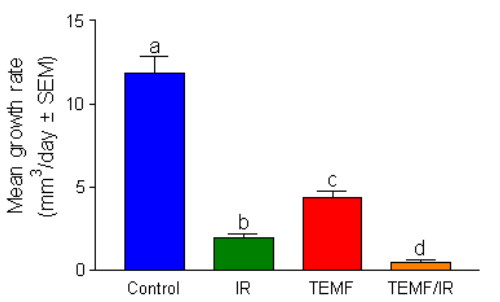
Mean ± SEM tumor growth rate data from day 7 to day 15 post IR therapy in each treatment group. ANOVA was used to determine any statistical differences in growth rates between treatment groups. Different letters demonstrate significant differences. Tumors in the untreated control group grew significantly faster than did tumors in the other three groups. Tumors in the IR/TEMF group had a growth rate significantly lower than that of the other three groups.

Results of an evaluation of the tumor growth rates from day 7 though day 15 post IR treatment are reported in Fig. 3. As illustrated in Fig. 3, the mean tumor growth rate of mice given neither IR nor TEMF treatment was significantly higher than the three groups given IR and TEMF therapy either alone or in combination. The group of mice given both IR and TEMF followed by daily TEMF had a mean tumor growth rate significantly lower than that of the other three groups. Clearly, the continued application of daily TEMF therapy following IR therapy had an additive inhibitory effect on the tumor growth rate during the 7 to 15 days after the course of IR therapy.

### Tumor vasculature

**Figure 4:**
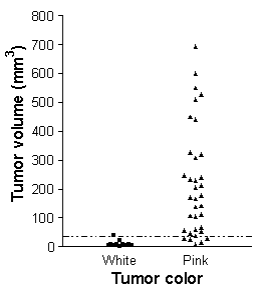
Relationship between color of the tumor as observed through the skin and the measured tumor volume. Tumors <35 mm^3 ^were mostly white while tumors >35 mm^3 ^were pink.

While making measurements on tumor volume, tumor color was observed through the skin of the nude mice. After recording tumor color and volume of each mouse, it became clear that smaller tumors were white while larger tumors were pink. Fig. 4 illustrates the relationship between tumor color and tumor volume. Almost all tumors less than 35 mm^3 ^were white whereas tumors greater than approximately 35 mm^3 ^were pink in color. Application of the pressure from one's finger to a pink tumor followed by the rapid removal of the finger pressure showed a rapid color change from white to pink.

**Figure 5:**
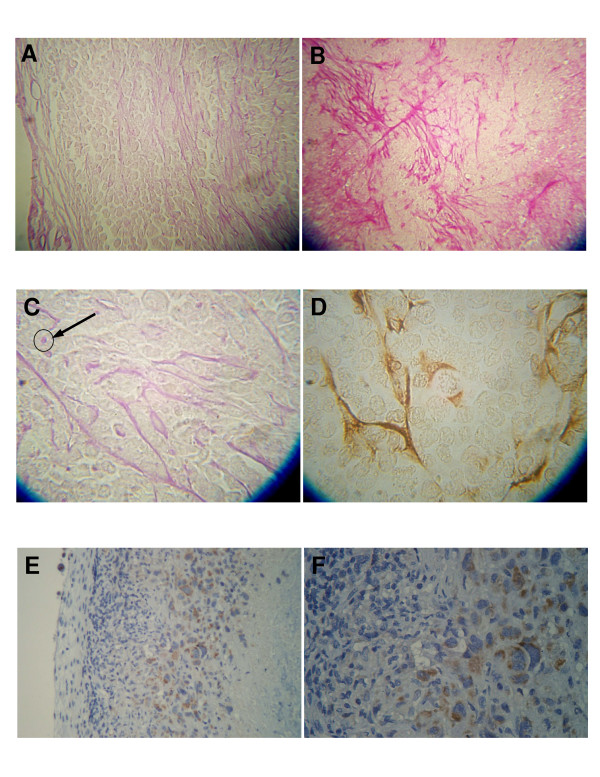
Photomicrographs illustrating the pattern of the tumor vascular network (A and B), of endothelial pseudopods stained with PAS (C) and with CD-31 specific endothelial markers (D), and of the localization of HIF-α (E and F). A, The tumor capsule (left) reveals blood vessels. The cortex under the capsule reveals no blood vessels and few endothelial pseudopods while the subcortical area to the right has more pseudopods. B, The subcortical area of the tumor reveals a small blood capillary with multiple endothelial pseudopods protruding at right angles into the tumor mass. C, At high magnification endothelial pseudopods are seen to branch and occasionally have a vacuole/lumen (arrow). D, The endothelial pseudopods react positively to the CD-31 specific endothelial marker. The CD-31 antibody identifies endothelial cells using the avidin-biotin peroxidase complex method. E, Viable cell area can be seen beneath tumor capsule (left). Necrotic area can be seen to the right. F, Enlarged subcortical area from E. In E the hypoxic area between the viable and the necrotic tissue is stained brown.

Histological examination of midsections of PAS stained tumors was done to assess tumor vascularization patterns. Tumors with a volume >35 mm^3 ^demonstrated a connective tissue capsule with blood vessels (Fig. 5A). At higher magnification the cortical area within about 100 μm of the capsule revealed few blood vessels while the area greater than 100 μm from the capsule showed evidence of considerable blood vessel and capillaries with many endothelial pseudopods extending away from the capillaries (Fig. 5B&5C). The general direction of the pseudopods was parallel to the tumor capsule surface. Immunohistochemical localization of CD-31, used as a specific marker of endothelium, demonstrated a positive reaction of pseudopods (Fig. 5D). Areas of tumor necrosis were observed, below the subcortical area (Fig. 5E&5F). Immunohistochemical localization of hypoxia-inducible factor 1-α (HIF) reveals the subcortical area of the tumor to contain HIF positive cells while the tumor capsule, cortex and necrotic areas of the tumor demonstrate no evidence of HIF. Thus, the area found to be HIF positive were enriched in endothelial pseudopods.

**Figure 6:**
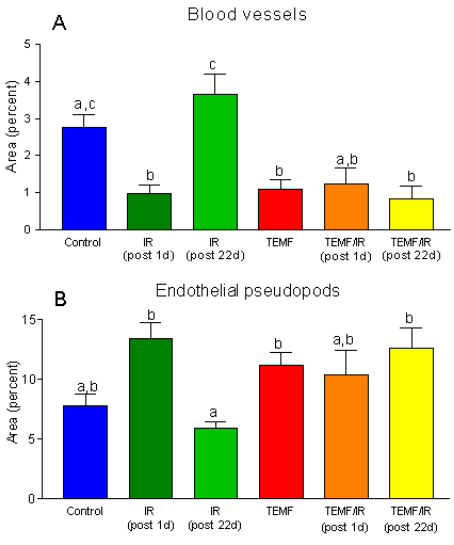
Quantification of changes in tumor vascularization between treatment groups. A and B, The percent of areas (volume density) of blood vessels and the percent of area of endothelial pseudopods were determined using an ocular grid intercept counting method. The mean ± SEM of each treatment group is graphed. Columns that do not share a common letter within a graph are significantly different (p < 0.01). The data indicate that gamma irradiation and EMF alone or in combination decreased the total area of blood vessels and increased the total area of pseudopods compared to the control. Data from the untreated mice (Control) and the EMF treated mice (TEMF) were pooled from mice from the early and from the late sacrifice because there was no significant time of sacrifice difference within these groups.

Ocular grid intercept couting was used to quantify tumor vascularization in 8 μm thick PAS stained histological sections of the tumors. The numbers of ocular grid intercepts were scored over the area of: blood vessels and capillaries, endothelial pseudopods and over the area with no indication of these structures. This method has been shown to be a usable measure of volume density occupied by recognizable structures. The results of the scoring of blood vessels and of endothelial pseudopods in the subcortical regions of the tumors are summarized in Fig. 6A&6B. Statistical analysis of the mean blood vessel volume density versus the pseudopod volume density reveals a significant exponential correlation coefficient of 0.966. Thus, as the blood vessel volume density decreased, the endothelial pseudopod volume density increased. As illustrated in Fig. 6A&6B, IR resulted in significantly reduced blood vessel volume density but a significantly increased volume density of pseudopods at one day after the last dose of irradiation, but the blood vessel volume density increased and the pseudopod volume density decreased to the level of the untreated control by 22 days after the last dose of irradiation. However, the groups of mice treated solely with EMF had significantly less blood vessel volume density and a significantly higher pseudopod volume density at one day after the end of IR. The low blood vessel volume density and the high pseudopods volume density in the tumors of the EMF treated mice remained the same at 22 days after the end of the IR treatment.

### Metastasis

**Figure 7:**
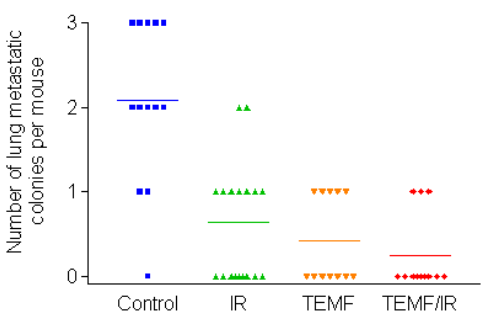
Metastasis of GFP-expressing human breast MDA MB231 cells from the site of inoculation in the inguinal mammary fat pad to lodge in the lungs of mice in each of the four treatment groups. The lungs were removed and smashed between microscopic slides and microcolonies of GFP cells were observed by epilumination using blue light and detected by presence of green fluorescence microcolonies observed using both 3.5× and 10× objective lens. The untreated mice had a significantly higher number of GFP positive microcolonies than the groups of mice that received gamma irradiation therapy or those mice that received EMF therapy. There were no other significant differences between groups in either mean number of microcolonies per lung or in incidence. Horizontal lines indicate mean values for each treatment group.

The number of lung metastatic colonies per mouse in each of the four treatment groups is summarized in Fig. 7. Both the mean number of colonies in the lungs per mouse as well as the incidence of metastasis was significantly higher in the untreated group than in the three groups treated with IR and/or EMF therapy. There were no significant differences between the three treatment groups in number or in incidence of lung metastatic colonies per mouse.

### Side Effects of Gamma Irradiation Therapy and of TEMF Therapy

Mice were euthanized at both one day and at 22 days after the last IR exposure. Data on liver and spleen weights are summarized in Table 1, and data on blood counts are summarized in Table 2. There were no statistically significant differences between treatment groups in mean liver weights at the earlier or later kills. On the other hand, the spleen weights of mice that were euthanized one day after their last exposure to IR were significantly less than the spleen weights of the non-gamma irradiated groups. The mean spleen weights of the IR mice recovered to the level of the untreated groups by 22 days after their last IR exposure. There was no significant loss of spleen weight at the time of early or at the time of late euthanasia due to TEMF therapy.

**Table 1:** Gamma Irradiation Therapy (IR) and Therapeutic Electromagnetic Field Therapy (TEMF) on Liver and Spleen Weight (Means ± SEM)

Therapy Group	*n*	Liver weight (grams)	Spleen weight (grams)
**Early**^1^			
Control	5	0.94 ± 0.04	0.107 ± 0.009
IR	5	0.87 ± 0.07	0.031 ± 0.004
TEMF	5	1.04 ± 0.05	0.110 ± 0.006
TEMF/IR	5	0.93 ± 0.04	0.033 ± 0.004
**Late**^2^			
Control	9	1.26 ± 0.07	0.166 ± 0.008
IR	20	1.17 ± 0.03	0.146 ± 0.008
TEMF	10	1.16 ± 0.05	0.145 ± 0.012
TEMF/IR	10	1.15 ± 0.05	0.151 ± 0.014

^1^Mice sacrificed one day after last irradiation treatment.

^2^Mice sacrificed 22 days after last irradiation treatment.

Results of Statistical Analyses:

1. Liver weight: No significant differences among treatment groups.

2. Spleen weight: At early kill, groups treated with gamma irradiation show significant (p < 0.01) loss of weight compared to groups not treated with gamma irradiation. In late kill, no significant differences were detected.

**Table 2:** Gamma Irradiation Therapy (IR) and Electromagnetic Field Therapy (TEMF) on Blood Counts(Means ± SEM)

Therapy Group	*n*	WBC × 10^3^/μL	RBC × 10^6^/μL	Platelets × 10^3^/μL	Micronuclei (%RBC)
**Early**^1^					
Control	4	2.91 ± 0.83	9.08 ± 0.07	584 ± 76	1.3 ± 0.04
IR	4	0.11 ± 0.01	7.50 ± 0.16	389 ± 17	1.2 ± 0.03
TEMF	5	2.89 ± 0.60	9.05 ± 0.12	505 ± 61	1.3 ± 0.04
TEMF/IR	4	0.23 ± 0.06	7.68 ± 0.13	443 ± 34	1.2 ± 0.03
**Late**^2^					
Control	8	1.85 ± 0.37	8.30 ± 0.14	551 ± 83	1.6 ± 0.06
IR	13	1.48 ± 0.332	7.06 ± 0.16	973 ± 98	0.7 ± 0.01
TEMF	11	1.15 ± 0.29	8.85 ± 0.22	613 ± 62	0.7 ± 0.01
TEMF/IR	10	1.07 ± 0.13	6.58 ± 0.11	1007 ± 141	0.6 ± 0.01

^1^Mice sacrificed one day after last irradiation treatment.

^2^Mice sacrificed 22 days after last irradiation treatment.

Results of Statistical Analyses:

1. At the end of irradiation therapy plus one day, gamma irradiation caused significant decreases in WBC, RBC, and platelet counts. No other significant differences at p < 0.01.

2. At the end of irradiation therapy plus 22 days, gamma irradiation caused significant decreases in WBC and RBC counts and a significant increase in platelet counts. No other significant differences at p < 0.01.

There was a significant decrease in WBC, RBC, and platelets counts attributed to gamma irradiation exposure at 1 day after the end of radiation treatment (Table 2). There was no significant difference in WBC, RBC, and platelets counts attributed to TEMF treatment. At 22 days after the end of the IR treatment, the WBC and RBC counts in the two IR groups were not quite as low but remained significantly lower than in the two non-irradiated groups while the IR groups had significantly higher platelet counts than any of the other groups. Clearly, platelet counts have made a significant compensatory rebound by 22 days after the end of IR therapy. Statistical analysis of micronuclei counts indicated that there was no significant genotoxic damage due to either treatment at either time of euthanasia.

**Figure 8:**
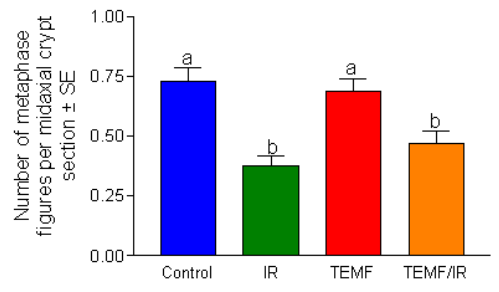
Number of metaphase figures per midaxial histological section of duodenal crypts. Column height indicates mean ± SEM. The data are from the mice sacrificed one day after the last gamma irradiation exposure. The columns that do not share a common letter are significantly different. Gamma irradiation but not EMF decreased the number of metaphase figures in the duodenal crypts.

Another measure of possible side effects was the scoring of mitotic activity in the duodenal crypts. Fig. 8 summarizes results of mitotic activity in the duodenal crypts of mice sacrificed one day after the end of the IR therapy regimen. The two groups of mice treated with IR 24 hours previously had significantly fewer metaphase figures per crypt than the non-treated group or to the group of mice treated solely with the course of TEMF therapy.

All mice treated with IR demonstrated a tan skin discoloration immediately after the 8-day course of IR therapy, but the tan skin color returned to the normal skin color by 22 days after the course of IR therapy was terminated. The skin color in the two non-irradiated groups of mice remained normal in appearance throughout the course of the study.

## Discussion

### On tumor vascularization

The immunohistochemical localization of hypoxia induced factor (HIF) in the tumors helped explain the vascular pattern of the encapsulated tumors. The HIF positive reactivity in the tumor was localized to the subcortical areas of the tumor in the same area found to have the most blood vessels, capillaries and pseudopods. These observations suggest that this subcortical area of the tumor is hypoxic leading to production of angiogenesis growth factors, which in turn acts on the host endothelial cells to sprout pseudopods. Sprouting of cultured endothelial cells in response to vascular endothelial growth factors is known to occur [4]. Feraud et al. also reported that this endothelial cell sprouting could be inhibited by several angiostatic agents. The presence of pseudopodal like structures in viable areas of a tumor immediately adjacent to necrotic areas of the tumor has been previously reported [5]. The lack of HIF reactivity in the cortical area beneath the well vascularized tumor capsule suggest this cortical area is not hypoxic and not in need of an extensive vasculature. It appears that the PSA staining of endothelial cell sprouting pseudopods in tumors gives quantifiable spatial information on where in the tumor that HIF and vascular endothelial growth factors are located.

### Action of IR and of EMF therapies

EMF therapy has been reported to slow tumor growth and tumor angiogenesis and to increase the survival time of tumor bearing mice [1]. An experiment was therefore designed to test if the combining of IR therapy and EMF therapy in a tumor bearing mouse model might give an addictive therapeutic index.

Over half of all cancer patients are given ionizing radiation (IR) during their course of treatment [6]. The interactions between IR and tumor vascularization are complex [7,8]. It has been shown that IR therapy is dependent on tumor vascularization and that IR therapy initially interferes with tumor vascularization leading to tumor hypoxia [9]. However hypoxic areas are radio-resistant until they can induce angiogenesis and re-oxygenation. Hypoxic areas of tumors produce HIF leading to production of angiogenesis growth factors, which stimulate angiogenesis to allow further tumor growth. This known sequence of events following an IR treatment of a tumorous patient has lead to the combined use of IR therapy coupled to use of an anti-angiogenesis agent. By blocking angiogenic factors, like VEGF, and by blocking the formation of new vessels in combination with IR therapy researchers have dramatically increased the efficacy of IR therapy in a number of tumor types [7-9]. The use of anti-angiogenic agents has proved useful in the treatment of cancer progression in most preclinical and clinical trials [10,11]. However the prevention of metastasis (Inoue et al.) by anti-angiogenic agents [11,12] has not gone unchallenged [[13] and see rebuttal by Kieran et al. [14]].

The experimental results indicate that either IR therapy or EMF therapy suppressed tumor growth. The IR therapy proved to be more effective at suppressing tumor growth than did the EMF therapy while the IR therapy but not the EMF therapy had harmful side effects. Both IR therapy and EMF therapy reduced blood vessel volume density within the tumor when measured one day after the end of the IR therapy but blood vessel volume density in tumors of the IR treated mice returned to pre IR treatment levels. The continual use of daily EMF therapy in the IR treated mice did suppress the return of blood vessel volume density within the tumor following IR therapy. The combination of both therapeutic modalities therefore led to a sustained and significant reduction in the extent of tumor blood vessel volume density.

That pseudopod volume density was relatively high at 22 day after IR in the group of mice that were on sustained daily EMF therapy suggests that major areas of these tumors were anoxic and thus were producing HIF and VEGF's resulting in the sprouting of the endothelial cell pseudopods. This indicates that these early events in the angiogenesis process were not suppressed by the continued use of EMF therapy. Because continued use of EMF did suppress tumor blood vessel volume density at 22 days after IR therapy the EMF therapy may be acting to suppress one of more of the later steps in the process of tumor vascularization such as: endothelial cell proliferation, lumen formation, generation of vascular tubes and initiation of blood flow [15].

As illustrated in Fig. 6A, the continued daily use of TEMF following the course of IR therapy suppressed tumor blood vessel volume density compared to mice given the IR therapy alone. The data on tumor growth rate, beginning a week after IR therapy, revealed that the continuation of TEMF therapy suppressed the mean tumor growth rate of the tumors compared to those of mice treated only with IR therapy. In fact, linear regression analysis of the data indicates that the slope of the tumor growth rate of the IR plus TEMF treated mice did not differ from a slope of zero. Thus, these tumor growth rate results indicate a significant treatment advantage for continuous daily TEMF therapy following a standard course of IR therapy.

Since the extent of tumor vascularization has been positively linked to the likelihood of tumor cell metastasis [12,16-18] it was anticipated that a decrease in the blood vessel volume density in the tumor, as observed in the experiments reported herein, would decrease the chance for tumor cells to metastasize to distant sites in the tumor bearing mouse model used in the study. Inspection of the incidence of metastasis observed in the lungs of human breast cancer xenographs revealed: that IR therapy alone reduced metastasis by 49%, that EMF therapy alone reduced metastasis by 54% while the combination of IR and EMF therapies when followed by daily EMF therapy resulted in a 73% reduction in incidence of metastasis to the lung. Although all three of these therapy regimens caused a significant decrease in incidence of metastasis of tumor cells to the lungs the greatest decrease, although not statistically significant, was with the combination of IR and EMF therapies.

In summary the results indicate that sustained EMF therapy by itself reduced: the extent of tumor blood vessel volume density, the tumor growth rate and tumor cell metastasis to the lung, without harmful side effects. TEMF therapy alone may therefore be a useful alternate therapy for cancer patients who decide not to undergo standard radiation or for chemotherapy. The IR therapy alone markedly reduced: the tumor growth rate, reduced tumor metastasis but only transitly reduced tumor blood vessel volume density. The IR therapy did however have harmful side effects. These side effects could be minimized by targeted IR exposure. The combination of IR therapy and continued TEMF therapy markedly suppressed the return of blood vessel volume density and tumor growth following IR therapy and resulted in the lowest incidence of tumor metastasis. Thus these findings support the ideas that TEMF of the type used in this experiment may be an effective antiangiogenic therapy and that a combination of an anti-vascular modality, such as TEMF, with IR therapy may enhance the therapeutic index of IR therapy for cancer.

## Materials and methods

### Animals

Female athymic nude mice (purchased from the Harlan Sprague Dawley, Inc., Indianapolis, IN), 6 weeks of age, were used for this study. The animals were housed under pathogen-free conditions and fed an AIN-76 semipurified diet slightly altered to contain 10% w/w corn oil.

### Cell Lines

Human breast cancer cell line MDA-MB-231 was obtained from the American Type Culture Collection. To determine the effect of the treatments on the metastatic potential of the MDA-MB-231 cells they were stably transfected with the enhanced GFP expression plasmid, pEGFP-N1 (Clontech Laboratories, Inc.). The expression of GFP allowed for detection of micrometastatic colonies on the whole flattened lungs with a fluorescence epilumination microscope. The cell lines were cultured in McCoys's 5A medium supplemented with pyruvate, vitamins, amino acids, antibiotics, and 10% fetal bovine serum [2]. HDMECs and the culture medium EGM-2 Mv were maintained at 37°C in a humidified incubator with 5% CO_2_.

### *In Vivo *Tumor Growth

The MDA-MB-231/GFP cells were harvested from exponential cultures and inoculated at 2 × 10^6 ^cells/inoculum in the inguinal mammary fat pad area of female athymic nude mice, 6 weeks of age. When the tumors grew to an average diameter of about 3 mm after 5 weeks, the mice were divided into 4 groups such that the mean and median of tumor volume of the four groups were closely matched. Growth of each xenograft was monitored by externally measuring tumors in three dimensions using digital calipers 3 times per week. Xenograft volume (V) was determined by the following equation: V = (L × W^2^) × 0.5, where L is the length and W is the width of a xenograft.

### Gamma Irradiation (IR) Therapy

Eighty tumor-bearing mice received a cumulative dose of 800 cGy of IR (200 cGy each third day for 4 cycles). The 200 cGy/day dosage is based on the results from a preliminary dose response study using the same mouse model and on the fact that 180 to 250 cGy/day is the commonly used acute dose for radiotherapy of humans. About 815 cGy whole body radiation is the LD50/30 for mice (50% lethal within 30 days). Mice were irradiated in a ^137^Cs Gamma Cell-40 Irradiator (Atomic Energy of Canada) facility in our Department of Radiology. Dosimetric analyses for this instrument is performed monthly for calculation of a precise 200 cGy exposure. Mice were transferred to a circular cage with individual compartments for each mouse during irradiation. Mice were euthanized at one day or 23 days after the last radiation treatment. The peripheral blood, duodenum, lungs, liver, spleen and tumor were harvested for analyses and the carcasses frozen in liquid nitrogen and stored at -20°C for later analysis of metastasis.

### Magnetic Field Therapy

A therapeutic electromagnetic field (TEMF) system having a proprietary signal devised by EMF Therapeutics, Inc. (Chattanooga, TN, USA) was used. The system generates a pulsating half sinewave magnetic field with a frequency of 120 pulses per second [1]. An ellipsoidal coil with 21" major axis and 14" minor axis was used to generate the magnetic field. In the experiment reported here, the magnetic flux density measured in the exposure chamber was 15 mT. This value of magnetic field flux density was chosen based upon our previous experience and data [1]. A thorough 3-D mapping of the magnetic filed was performed for the entire space covered by the coil. The flux density of the magnetic field in the exposure chamber (25 cm long, 10 cm wide and 13 cm high) was consistent within the entire volume of the chamber. The walls of the exposure chamber were perforated to allow air exchange and to limit the temperature change inside the exposure chamber during the TEMF treatment was less than 1°C.

### Euthanasia and Tissue Handling

Mice were deeply anesthetized using a ketamine/rompun mixture prepared by the UTHSCSA Laboratory Animal veterinarian, cervically dislocated, then were exsanguinated by cardiac puncture. Blood was collected into an EDTA containing microtube for complete blood counts.

The tumor, duodenum, spleen and liver were removed at the time of euthanasia while the carcass (including the lungs) was frozen and stored at -20°F for later analyses. Sections of the duodenum, the tumor, and a lobe of liver were fixed in Omni Fix II (Mt. Vernon N.Y.) and paraffin embedded. Embedded tissues were cut 4 μm or 8 μm thick, cut sections were placed on microscope slides then deparafinized and stained with hematoxylin and eosin or with periodic acid-Schiff (PAS) for morphological analysis. Additional sections were prepared for immunohistochemistry.

### Metastasis studies

Lungs were removed from the thawed carcass to examine any spontaneous metastasis. The lungs were placed on a 25 × 75 mm microscope slide then smashed with a second slide to an area of about 100 mm^2^. The smashed lungs were secured in the flattened condition by rapping Magic tape (Scotch) around each end of the smashed lung preparation. The GFP-expressing metastatic cancer cell colonies, if any, were identified and counted using a Zeiss fluorescence microscope (TE-200) with a 3.5× and a 10× objective. GFP micrometastatic sites with three or more cells were scored as positive. No large metastatic sites were observed in the lungs.

### Tumor Vascularity

To determine the effect of treatment on tumor angiogenesis, we measured the vascularity of excised tumors. Tumor tissues were fixed and imbedded in paraffin. Mid tumor sections of 8 μm were cut from the embedded tissue and stained with periodic acid Schiff (PAS). Sections were examined by light microscopy. CD31 immunostaining for mouse blood vessels was performed by incubating tumor sections with a rat antimouse CD-31 (PECAM-1) monoclonal antibody (PharMingen) at 5 μg/ml for 30 min at 37°C. Sections were then incubated with a biotin-labeled goat antirat IgG (Zymed; 1:200 dilution) for 30 min at room temperature, followed by ABC reagent kit (Vector Laboratories) for 30 min at room temperature. Color reaction was performed with 3, 3'-diaminobenzidine (Vector Laboratories) and counterstained with hematoxylin. Hypoxia-inducible factor-1 alpha immunohistochemistry was done following the instruction for antigen retrieval (Biogenex protocol) and iso-IHC (inno-Genex Mouse-on-mouse iso-IHC kit) with these changes: Dewaxing was with 3 changes of xylene, 10 minutes/change. Rehydration in 100%, 90%, and 70% ethanol, 10 minutes each. Initial dilution of the antibody was 1:200. Antibody: Stressgen anti-HIF-1 alpha, product # OSA-601. All sections were coded, then treated as above and the extent of blood vessels, endothelial cell pseudopods and total area volume density was scored using an ocular grid. The number of grid line intercepts over blood vessels and endothelial pseudopods gave a measure of the total volume density of these structures.

## Assays Performed

### Complete and differential blood counts

A blood cell counter with veterinary pack was used for counts of red cells, white cells and platelets in EDTA anticoagulated blood of the mice. Our Laboratory Animal Resources division performed this test.

### Peripheral blood micronuclei

At least 1000 acridine orange stained cells were counted and the percentage of erythrocytes containing micronuclei was determined [3].

### Histological analyses of duodenum

Fixed specimens of duodenum were trimmed, processed and oriented for paraffin embedding. Four μm thick sections of the paraffin blocks were mounted on slides. Complete midaxially sectioned crypts on H&E stained slides were selected for analyses. Complete crypts were defined as those with: 1) the crypt base at the muscularis mucosa, 2) an open lumen from mouth to base and 3) a single column of epithelial cells up each side of the crypt. The numbers of metaphase figures per midaxial crypt section was counted for 10 crypts of each mouse.

### Statistical analyses

SAS computer software was used for statistical analyses. Tests for normality (basic statistics) was used on each data set. One way and two way analyses of variance followed by Student-Newman-Keuls multiple range tests, as appropriate, was used to determine if there were statistically significant (p ≤ 0.05) differences in any measured parameter due to the therapies. Tumor volume change for each tumor was determined using least squares linear regression analysis of tumor volume using Prism™ (Graphpad Software, Inc.). Differences in the proportions of mice with lung metastatic sites were assessed using the Fischer exact test analyses.

## The abbreviations used

GFP, green fluorescent protein; TEMF, therapeutic electromagnetic field; PAS, periodic acid Schiff; HIF, hypoxia inducible factor 1- alpha; CD-31, monoclonal antibody against endothelial cells; IR, ionizing irradiation

## Conflict of Interest

The author(s) declare that they have no competing interests.

## References

[B1] William CD, Markov MS, Hardman WE, Cameron IL (2001). Therapeutic electromagnetic field effects on angiogenesis and tumor growth. Anticancer Res.

[B2] Bandyopadhyay A, López-Casillas F, Malik SN, Montiel JL, Mendoz V, Yang J, Sun LZ (2002). Antitumor activity of a recombinant soluble betaglycan in human cancer xenograft. Can Res.

[B3] Vijayalaxmi, Frei MR, Dusch SJ, Gue V, Meltz ML, Jauchem JR (1997). Frequency of micronuclei in the peripheral blood and bone marrow of cancer-prone mice chronically exposed to 2450 MHz radiofrequency radiation. Radiat Res.

[B4] Feraud O, Cao Y, Vittet D (2001). Embryonic stem cell-derived embryoid bodies development in collagen gels recapitulates sprouting angiogenesis. Lab Invest.

[B5] Yang M, Li L, Jiang P, Moossam AR, Penman S, Hoffman RM (2003). Dual-color fluorescence imaging distinguishes tumor cells from induced host angiogenic vessels and stromal cells. Proc Natl Acad Sci USA.

[B6] Owen JB, Coia LR, Hanks (1992). Recent patterns of growth in radiation therapy facilities in the United States: a pattern of care study report. Int J Radiat Onc Bio Phys.

[B7] Wachsberger P, Burd R, Dicken AP (2003). Tumor response to ionizing radiation combined with antiangiogenesis or vascular targeting agents: exploring mechanisms of interaction. Clin Can Res.

[B8] Wachsberger P, Burd R, Dicken AP Ionizing radiation and anti-vascular therapy: exploring mechanisms of tumor response. Clin Can Res.

[B9] Gorski DH, Beckett MA, Jasowiak NT, Calvin DP, Mauceri HJ, Salloum RM, Seetharam S, Koons A, Hari DM, Kufe DW, Weichselbaum RR (1999). Blockade of the vascular endothelial growth factor stress response increases antitumor effects of ionizing radiation. Can Res.

[B10] Weidner N (2002). New paradigm for vessel intravasation by tumor cells. Amer J Path.

[B11] Inoue K, Chikayawa M, Fukatas, Yoshikawa C, Shuin T (2002). Frequent administration of angiogenesis inhibitor TNP-470 (AGM-1470) at an optimal biological dose inhibits tumor growth and metastasis of metastatic human transitional cell carcinoma in the urinary bladder. Clin Can Res.

[B12] Crepin M, DiBenedetto M, Bagheri-Yarmand R, Starzec A, Vassy R, Perret G (2003). Prevention of breast tumor angiogenesis and metastasis by cytostatic molecules in relevant mouse models. Breast Can Res.

[B13] Steeg PS (2003). Angiogenesis inhibitors: motivators of metastasis?. Nat Med.

[B14] Kieran MW, Folkman J, Haymach J (2003). Angiogenesis inhibitors and hypoxia. Nat Med.

[B15] Ribatti D, Vacca A, De Falco G, Roccaro A, Roncali L, Dammacco F (2001). Angiogenesis, angiogenic factors expression and hematological malignances. Anticancer Res.

[B16] Jaeger TM, Weidner N, Chew K, Moore DH, Kerschmann RL, Waldman FM, Carroll PR (1995). Tumor angiogenesis correlates with lymph node metastases in invasive bladder cancer. J Urol.

[B17] Weidner N, Semple JP, Welch WR (1991). Tumor angiogenesis and metastasis-correlation in invasive breast cancer carcinoma. N Eng J Med.

[B18] Leek RD (2001). The prognostic role of angiogenesis in breast cancer. Anticancer Res.

